# A Possible Mechanism for the Suppression of *Plasmodium berghei* Development in the Mosquito *Anopheles gambiae* by the Microsporidian *Vavraia culicis*

**DOI:** 10.1371/journal.pone.0004676

**Published:** 2009-03-11

**Authors:** Irka Bargielowski, Jacob C. Koella

**Affiliations:** Division of Biology, Faculty of Natural Sciences, Imperial College London, London, United Kingdom; University of Liverpool, United Kingdom

## Abstract

**Background:**

Microsporidian parasites of mosquitoes offer a possible way of controlling malaria, as they impede the development of *Plasmodium* parasites within the mosquito. The mechanism involved in this interference process is unknown.

**Methodology:**

We evaluated the possibility that larval infection by a microsporidian primes the immune system of adult mosquitoes in a way that enables a more effective anti-*Plasmodium* response. To do so, we infected 2-day old larvae of the mosquito *Anopheles gambiae* with one of 4 isolates of the microsporidian *Vavraia culicis* and reared one group as an uninfected control. Within each treatment, we fed half the adult females on a mix of *P. berghei* ookinetes and blood and inoculated the other half with a negatively charged CM-25 Sephadex bead to evaluate the mosquitoes' melanisation response.

**Conclusions:**

The microsporidian-infected mosquitoes were less likely to harbour oocysts (58.5% vs. 81.8%), harboured fewer oocysts (8.9 oocysts vs. 20.7 oocysts) if the malaria parasite did develop and melanised the Sephadex bead to a greater degree (73% vs. 35%) than the controls. While the isolates differed in the number of oocysts and in the melanisation response, the stimulation of the immune response was not correlated with either measure of malaria development. Nevertheless, the consistent difference between microsporidian-infected and –uninfected mosquitoes — more effective melanisation and less successful infection by malaria — suggests that microsporidians impede the development of malaria by priming the mosquito's immune system.

## Introduction

Microsporidian parasites of mosquitoes offer a possibility of effective malaria control, as they target several factors that determine the epidemiology of malaria: they reduce mosquito populations by increasing larval and pupal mortality and by decreasing fecundity [1]–[2], they reduce the lifespan of adult mosquitoes [3]–[4] and they decrease their biting rate [5]. Moreover, several microsporidian species interfere with the development of malaria parasites in the mosquito vector [6]–[9].

The mechanism involved in achieving this interference is unknown. Possibilities include that the microsporidians use resources required for the development of malaria and that microsporidians block molecular targets used by malaria parasites to invade the mosquito's midgut. In this study we consider the possibility that a microsporidian infection primes the mosquito's immune system in a way that helps it to defend itself against a later infection by *Plasmodium*. Unlike the adaptive immune system of vertebrates, which possesses antigen-specific lymphocyte populations and memory cells capable of recognising and dealing with previously encountered infections, the innate immune system of invertebrates lacks such memory cells. Nevertheless, pre-exposure to infective agents primes the invertebrates' immune system in such a way that it is more effective at dealing with subsequent infection. This non-specific memory, conferred by the upregulation of generic defence mechanisms is described as immune priming and is involved in several invertebrate-parasite interactions (reviewed in [10]). For example, challenging the beetle *Tenebrio molitor* with a bacteria-derived elicitor (LPS) decreases the success of a subsequent infection by a fungus (*Metarhizium anisopliae*) [11]. Immune-priming can also affect the development of malaria parasites. Thus, in a series of experiments, an earlier challenge by bacteria reduced the prevalence of malaria infection in mosquitoes [12]–[17], and mosquitoes treated with antibiotics expressed their immune genes to a lesser degree and were more susceptible to *Plasmodium* infection than untreated ones [18]–[19].

We investigate the role of immune-priming in the interaction between microsporidians and malaria by exposing larvae of the mosquito *Anopheles gambiae* to the infective spores of the microsporidian *Vavraia culicis* and testing the adult females for their immune response and for the success of infection by the malaria parasite *Plasmodium berghei*. We pose three questions: (i) Does *V. culicis* stimulate the immune system? (ii) Does the parasite suppress the development of *P. berghei*? (iii) Do isolates of *V. culicis* have similar effects and, in particular, are their effects on the mosquito's immune response and the development of malaria correlated?

Our measure of the immune response was the degree to which a CM-25 Sephadex bead injected into the mosquito's thorax was melanised. As the melanisation response is genetically correlated (i.e. shares part of its genetic basis) with the antibacterial response (at least in terms of the phenotypic outcome: the extent to which bacteria are cleared) [20], our measure covers several aspects of the immune system. Thus, we here consider the melanisation response as an indicator of the mosquito's general immune reaction to *Vavraia*, rather than a response that specifically clears malaria parasites. Indeed, anti-malaria responses appear to be associated with the mosquito's antimicrobial peptide system rather than a melanisation response [21].

## Results

We reared 1200 larvae individually in 12-well plates, in two consecutive blocks of 600 mosquitoes. 240 of these were uninfected controls, the others were infected with one of 4 microsporidian isolates. Out of the 701 mosquitoes that survived to adulthood, 327 were female and lived long enough to be used in the experiments. The numbers of females within treatments (59 in the control treatment; 60, 64, 70 and 74 for the four isolates) were similar (χ^2^ = 3.50, p = 0.478) and blocking had no effect on survival, so that our results were not biased because of larval mortality due to the microsporidian.

**Figure 1:**
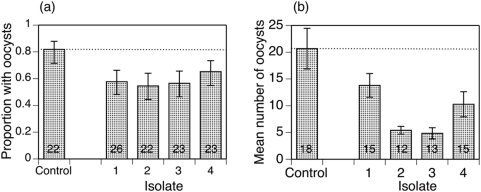
Success of infection by *P. berghei* in control mosquitoes and mosquitoes infected by one of four isolates of *V. culicis*. (a) The proportion of mosquitoes that harboured at least one oocyst 10 days after blood feeding. (b) The mean number of oocysts in mosquitoes with at least one oocyst. In both panels, the vertical lines show the standard errors of the estimates, the horizontal, dotted lines show the means of the controls, and the numbers in the bars indicate the number of mosquitoes analyzed. The isolates are numbered in order of increasing melanisation efficacy (see Figure 2).

Of the 159 adult females exposed to the *Plasmodium*-infected blood meal, 119 (75%) took a full blood meal and survived the 10 days up to being dissected. There was no difference in feeding success between uninfected control mosquitoes (75.4%) and microsporidian-infected mosquitoes (74%) (χ^2^ = 0.03, p = 0.860), but the proportion of mosquitoes that fed did depend on the microsporidian isolate that infected them (χ^2^ = 7.55, p = 0.056), ranging from 64% to 87%. Of the 116 fed mosquitoes, 73 harboured oocysts 10 days later. Blocking had no effect on the feeding efficiency of the mosquitoes, except for treatment with isolate 3, where more mosquitoes from block one took a blood meal than block two (χ^2^ = 6.046, p = 0.0139). Microsporidian-infected mosquitoes were less likely (58.5%) to harbour oocysts than microsporidian-uninfected controls (81.8%) (χ^2^ = 4.40, p = 0.036) (Fig. 1a). The effect of the microsporidian isolate on the proportion of mosquitoes with oocysts ranged from 54.5% to 65.2%, but this difference was far from statistically significant (χ^2^ = 0.65, p = 0.885). The mean number of oocysts in the 73 mosquitoes with at least 1 oocyst was 11.9, ranging from 4.8 to 20.7 among the five treatments (Fig. 1b; analysis of square root of oocyst number: F_5,67_ = 7.82, p<0.001). Block had no effect on the number of oocysts harboured by mosquitoes in each treatment group. Mosquitoes infected by a microsporidian harboured an average of 8.9 oocysts; controls harboured 20.7 oocysts (F_1,69_ = 30.93, p<0.001). The mean number of oocysts in mosquitoes infected by different microsporidian isolates ranged from 4.8 to 13.8 (F_1,47_ = 5.45, p = 0.003).

**Figure 2:**
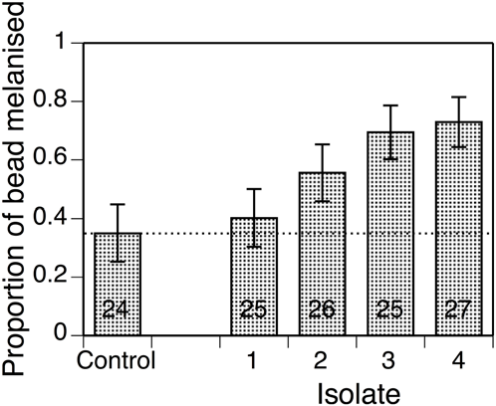
Efficacy of melanization response in control mosquitoes and mosquitoes infected by one of four isolates of *V. culicis*. Each point represents the mean proportion of a Sephadex bead melanized by a mosquito. The vertical lines represent the standard errors of the means and the horizontal, dotted line shows the mean of the controls. Again, the numbers in the bars indicate the number of mosquitoes sampled.

Of the 168 adult females inoculated with CM-25 Sephadex beads, 138 (80%) survived. Overall, the five treatments did not differ in their survival (χ^2^ = 4.33, p = 0.363), though there was a slight difference in survival between blocks one and two for mosquitoes infected with isolate 4 (χ^2^ = 3.873, p = 0.0491) and 1 (χ^2^ = 3.949, p = 0.0469). In eleven mosquitoes we could not find the bead, so that we analyzed 127 beads. The degree to which a bead was melanised ranged from 35% in the uninfected control mosquitoes to 73% in the mosquitoes infected with one of the microsporidian isolates (Fig. 2). Infection by any microsporidian isolate led to a stronger melanisation response than in uninfected controls (F_1,123_ = 11.47, p<0.001), and the four isolates differed in the degree to which they stimulated the melanisation response (F_3,95_ = 2.78 p = 0.004).

**Figure 3:**
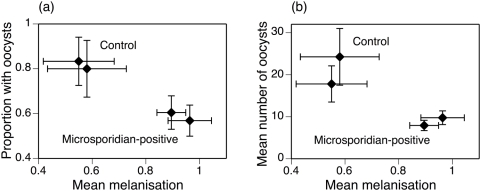
The association between the melanization response and the success of infection by *P. berghei*. Each point shows the mean within a block of the control or the microsporidian-infected mosquitoes (where the four isolates were pooled), and the horizontal and vertical lines show the standard errors of the estimates. (a) Association between the melanization response and the proportion of mosquitoes harbouring at least one oocyst 10 days after bloodfeeding. (b) Association between the melanization response and the mean number of oocysts.

Thus, larval infection by *V. culicis* enhanced the melanisation response in the adults and decreased the likelihood and intensity of infection by *P. berghei* (Fig. 3). In contrast to this clear association, there was no correlation within the microsporidian-infected mosquitoes between the melanisation response induced by an isolate and either the likelihood (F_1,5_ = 0.64, p = 0.459) or the intensity of infection (F_1,5_ = 1.63, p = 0.258) by malaria. Note, however, that with only four isolates in the experiment, the power to detect these correlations was weak (likelihood of infection: power = 0.1; intensity of infection: power = 0.18).

## Discussion

Larval infection by any of four isolates of the microsporidian *V. culicis* enhanced the melanisation response of adult *A. gambiae* and reduced the likelihood and intensity of infection by *P. berghei*. These results suggest that microsporidians impede the development of malaria in their mosquito vector by priming its immune system.

That *V. culicis* enhanced the immune (and in particular the melanisation) response of adult *A. gambiae* is far from trivial. Indeed, as *V. culicis* depletes the resources of infected larvae, leaving them with fewer lipids, sugars and glycogen reserves than uninfected individuals [22], the body condition of emerging adults is worse and one should therefore expect their immune response to be weak. This, indeed, seems to be the case in *Tenebrio molitor*, where infection by an (unnamed) microsporidian does not enhance the immune response, and in particular does not stimulate or enhance immune system parameters associated with melanisation [23]. In contrast, and similar to our study, in a proteomic study of *V. culicis* in another mosquito, *Aedes aegypti*, the antibacterial response was stimulated up to 15 days after infection [24]. Thus, both the melanisation pathway and the anti-*Plasmodium* response are primed, suggesting that either both these pathways are used in attempts to clear *V.culicis*, or that *V.culicis* infection leads to a general priming of the entire immune system.

That *V. culicis* impeded the development of malaria in its mosquito vector corroborates several studies, in which microsporidian infection reduced the proportion of mosquitoes developing oocysts and the number of oocysts [4], [9] and decreased the density [4] and quality [7] of sporozoites. This suppression appears to be a general characteristic of microsporidian infection, as similar results were found in all studied systems, including non-human malaria (e.g. *P. yoelii* [4]) as well as *P. falciparum* developing in *A. stephensi* [3] or the main African vector, *A. gambiae* [6]. What this study adds is that isolates impede development to different degrees, most likely caused by differences in their genetic make-up. This divergence is reflected in the microsporidian's and mosquito's phenotypic traits, e.g. the intensity of microsporidian infection, the mortality of larvae and pupae and the longevity of adults (Lorenz and Koella, unpublished data). Further experiments will investigate whether these traits are correlated with the mosquito's immune response and its reaction to malaria.

Both responses to microsporidian infection — more effective melanisation of a Sephadex bead and less successful infection by malaria — differed among the four microsporidian isolates. While it would be interesting to evaluate whether this variation is due to genetic differences among the isolates or other differences; e.g. maternal effects, this is not within the scope of this study. More importantly, for each isolate, microsporidian infection enhanced the melanisation response (and perhaps other immune responses) and impeded development of malaria, which suggests immune-priming as a mechanism. Stronger support would have been a negative correlation between enhanced melanisation by microsporidian isolates and interference with malaria by the same isolate, which we did not observe. However; one should not over-interpret this lack of association. First, the power to detect any such correlation was low, as we used only four isolates and the variation of the likelihood of malaria infection among the mosquitoes infected by the four isolates was low. Second, it is *a prioiri* not clear that an association should exist. A certain level of immune capability, for example, may eliminate the *Plasmodium* parasite or reduce its numbers. Beyond this threshold any increase in immune capability may have little effect on malarial infection, yet still be evident in the increasing melanisation response.

Another possible mechanism for the interference of microsporidian infection with *Plasmodium* might be competition for healthy gut cells. As microsporidians are intracellular parasites a possible immune response would be to shut down the cell through apoptosis, thus killing the microsporidian within. *Plasmodium* parasites, on the other hand, must pass through the midgut to form oocysts. It is therefore conceivable, that at this stage in the development process *Plasmodium* parasites that enter microsporidian-infected cells may be killed through apoptosis. Alternatively, *Plasmodium* parasites may not be able to develop in the presence of microsporidians, because of competition for a resource required by both parasites. It is possible that a more definitive answer to this question could have been reached by including a second set of controls in the experiment. Immune priming was only achieved with concurrent microsporidian infection, therefore a non-infective treatment such as lipopolysaccharide injections could have been used to induce non-infectious immune priming, allowing the comparison between microsporidian presence and absence to be made. However, these two possibilities do not explain the increased melanisation response associated with less effective infection by malaria. Therefore, immune priming remains the most likely explanation.

Further support for our suggestion that microsporidians impede the development of malaria by priming the immune response comes from a previous study, showing that microsporidian infection leads to an enhanced antibacterial response [24]. This not only corroborates the idea mentioned in the introduction — that the melanisation response reflects more general immune priming — but may be a direct immunological mechanism, as antimicrobial responses appear to be largely responsible for the clearing of malaria parasites [21]. Thus, immune-priming appears to stimulate the mosquito's immune system and prepare it for subsequent *Plasmodium* challenge, thus making the infected mosquito partially refractory to malarial infection. A similar immune system boost occurs in mosquitoes following bacterial challenge. If mosquitoes are infected with bacteria before being fed on malarious blood, they are less likely to be infected my malaria than if they are not infected by the bacteria [12]–[17], whereas mosquitoes treated with antibiotics display lower expression of immune genes and are more susceptible to *Plasmodium* infection [18]–[19].

Overall, our results suggest that microsporidians prime the immune response of mosquitoes in a way that impedes the development of malaria parasites.

## Materials and Methods

### Experimental design

The experiment was conducted in 2 blocks (2 consecutive clutches, 1 week apart), each comprising 600 mosquitoes. We exposed 120 larvae per block to one of four isolates of *V. culicis* and left 120 larvae uninfected. We divided the surviving females 3–5 days after emerging into two groups of equal numbers per treatment. One group was infected by *P. berghei* and the other group was inoculated with negatively charged CM-25 Sephadex beads. The number of oocysts ten days after infection was used as a measure of the success of malaria infection and the percentage of a bead covered by melanin 24 hours after inoculation was used as a measure of the efficacy of the melanisation response.

### Mosquito rearing

We used the G3 strain of *Anopheles gambiae* sensu stricto (Diptera: Culicidae), obtained from G. Christophides (Imperial College). Mosquitoes were reared at a temperature of 26 (+/−1) °C and 70 (+/−5) % relative humidity with a 12 h∶12 h light/dark cycle. Larvae were reared individually in 2 ml of de-ionised water in 12-well trays and fed with a standard amount of TetraMin Baby fish food (day 1: 0.06 mg, day 2: 0.12 mg, day 3: 0.24 mg, day 4: 0.36 mg, day 5: 0.48 mg, days 6 and later: 0.6 mg/individual).

### Infection by Vavraia culicis

The microsporidian *Vavraia culicis*, first identified in *Culex pipiens* in central Europe, infects several genera of mosquitoes including *Anopheles*, *Culex* and *Aedes* [25]. After ingestion by the mosquito larvae, the spores germinate and infect the host's gut cells. After undergoing a series of developmental stages, the parasite produces a new round of infectious spores several days (depending on conditions) after infection. The *V. culicis* spores used in this study were provided by J.J. Becnel (USDA Gainesville, USA), who had maintained the parasite on a laboratory colony of *Aedes aegypti*.

#### Isolates

Spores harboured by *Ae. aegypti* were used to infect a cohort of *Anopheles* larvae. We harvested the spores of each individual by grinding it in de-ionised water. We selected 20 samples with no apparent bacterial infection and at least 60000 spores per individual and maintained each of these isolates for several generations in groups of 50 *A. gambiae*. The isolates differ in several traits, e.g. infectivity of the parasites and longevity of infected adult mosquitoes (L. Lorenz, unpublished data). As each isolate was initiated with a different sample of the existing genotypes, the twenty isolates are likely to differ genetically, although the observed differences among isolates may also be due to other mechanisms, e.g. maternal effects. For our experiment we chose 4 of the 20 available isolates. Isolate 1 has low infection rates and reduces the lifespan of adults only slightly. Isolates 2, 3 and 4 are more virulent: they give high spore counts, reduce adult life expectancy considerably, and decrease the weight of adults (L. Lorenz, unpublished data).

#### Experimental procedure

The four *V. culicis* isolates were the product of repeated infections of at least four mosquito generations. We obtained the spores by homogenizing infected *A. gambiae* in de-ionised water. They were counted under a microscope (400× magnification) with a haemacytometer.

We exposed 2-day old *A. gambiae* larvae to *V. culicis* by adding 20000 spores in 1 ml of de-ionised water to each well; unexposed larvae received 1 ml of de-ionised water. From other experiments, we know that this concentration of spores generally gives close to 100% infection success.

The *A. gambiae* larvae were reared individually on 12-well plates (3 rows×4 columns). Each row was allocated one treatment, and 5 rows of 2 adjacent plates received the treatments in the order: controls – isolate 4 – isolate 1 – isolate 3 – isolate 2 until all 600 wells per block were treated. Pupae were moved to water-filled cups in mosquito cages (1 cage per isolate) and left to emerge. The adults had unlimited access to a 6% glucose solution.

### Infection by Plasmodium berghei

*Plasmodium berghei* is one of four species of malaria that have been described in murine rodents of West Africa. We exposed mosquitoes to ookinetes created *ex vivo*, as this gives the most reliable infections in mosquitoes (B. Sinden, pers. comm.). Each group of mosquitoes (the 5 treatments per block) was fed on two membrane feeders so that differences among groups could be attributed to the treatment rather than the feeders. The ookinetes were produced by staff of R. Sinden's lab of Imperial College London. We obtained the blood of uninfected mice (after injecting 300 µl Hypnorm (Janssen)) with a syringe and 21 gauge needle containing 500 µl of 200 units/ml heparin. The culture was incubated for 24 h at 19°C. We centrifuged the ookinete culture at 500 g for 10 minutes at 19°C, removed the supernatant and counted the remaining ookinetes with a microscope (400× magnification) and haemacytometer. We added blood obtained from uninfected mice to achieve a concentration of 800 ookinetes/µl and injected the mixture into the membrane feeders, which had been preheated to 37°C with a water bath (Julabu Labortechnik GmbH (ED GB)) and covered with Parafilm “M” (Pechiney Plastic Packaging). Mosquitoes were fed for 20–30 minutes. Mosquitoes that were not fully fed were discarded. Fully fed mosquitoes were kept at 19°C for ten days. We then mounted their midguts on slides and counted the oocysts under a microscope at 400× magnification.

### Melanisation assay

The degree to which Sephadex beads were melanised was used as a measure of immunity.

The beads range from 40–120 µm in diameter, of which we selected the smallest ones (estimated to range from 40 to 60 µm) visually. The beads were rehydrated in saline solution containing 1.3 mM NaCl, 0.5 mM KCl, 0.2 mM CaCl2 and 0.001% methyl green (pH 6.8) [26]–[27]. One bead was injected into the thorax of each mosquito (which had been briefly chilled on ice) with at most 0.3 µl of saline solution. The mosquitoes were then kept in falcon tubes lined with damp filter paper and supplied with cotton soaked in a 6% glucose solution. After 24 hours, the mosquitoes that were able to fly were dissected in a mixture of saline solution and 0.01% methyl green [27]. The percentage of the beads covered with melanin was estimated visually with a dissection microscope. Previous studies have shown that the variance of repeated estimates of a single bead is much less than the variance among beads (Koella, unpublished).

### Statistical analyses

Statistical analyses were performed with JMP version 6.0 (http://www.jmpdiscovery.com).

To control for possible biases, we analysed the likelihood that a mosquito survived to become an adult, blood-fed and survived the infection by malaria or survived the bead injection with logistic analyses. The likelihood of a successful malaria infection (i.e. the likelihood that a mosquito harboured at least one oocyst) was examined with a logistic analysis. The number of oocysts was analysed only in the mosquitoes where the malaria-infection was successful (i.e. where at least one oocyst was found). The square-root of the number of oocysts was evaluated with an ANOVA and gave normally distributed residuals. The proportion of a bead that was melanised was arc-sine transformed and analysed with an ANOVA. Each analysis included block as a nominal factor. For each trait, we tested for a difference between control and microsporidian-infected mosquitoes and for a difference among the microsporidian isolates with two separate analyses. We estimated the association between melanisation and the two measures of malaria infection by calculating the correlation of the average values per treatment and block, using block as a fixed factor in the analysis of covariance.
